# STING contributes to the inflammation and proliferation of *Staphylococcus aureus* via mitochondrial reactive oxygen species–hypoxic inducible factor 1α axis in epithelial cells

**DOI:** 10.1128/iai.00138-25

**Published:** 2025-05-19

**Authors:** Xing Gao, Binfeng Wu, Yawei Qiu, Shiyuan Feng, Jinqiu Zhang, Jinfeng Miao

**Affiliations:** 1 Ministry of Education Joint International Research Laboratory of Animal Health and Food Safety, College of Veterinary Medicine, Nanjing Agricultural University 261674, Nanjing, China; 2 Sanya Research Institute, Nanjing Agricultural University 663035, Sanya, China; 3 Institute of Veterinary Immunology & Engineering, Jiangsu Academy of Agricultural Sciences 117941, Nanjing, China; 4 Jiangsu Key Laboratory for Food Quality and Safety-State Key Laboratory Cultivation Base, Ministry of Science and Technology, Nanjing, China; St Jude Children's Research Hospital, Memphis, Tennessee, USA

**Keywords:** STING, mastitis, mROS-HIF1α, glycolysis, *Staphylococcus aureus*

## Abstract

*Staphylococcus aureus* infection poses a serious threat to the dairy industry and public health safety. The stimulator of interferon gene (STING) signaling pathway has been well established as effective in defending against viral infections. However, the role of STING is controversial during bacterial infections. Herein, we provide an insight into the role of STING during *S. aureus* infection. Our data revealed that the STING signaling pathway was activated in *S. aureus*-infected cells. *In vitro* investigations demonstrated that inhibiting STING reduced inflammation, hypoxia-inducible factor-1 alpha (HIF1α) expression, and mitochondrial reactive oxygen species (mROS) production. Interestingly, blocking HIF1α eliminated the escalation of inflammation associated with STING. Additionally, suppressing mROS production significantly reduced HIF1α expression and inflammation levels, while elevating mROS had the opposite effect. These results indicate that STING promoted inflammation through the mROS-HIF1α pathway. Given that glycolysis is driven by HIF1α, we investigated the role of glycolysis during infection. As expected, STING-elevated inflammation was linked with HIF1α-driven glycolysis. In terms of pathogenesis, STING contributed to *S. aureus* proliferation within cells and mouse mammary glands. Collectively, our findings demonstrate that STING facilitates infection via the mROS-HIF1α-glycolysis axis, highlighting its potential as a promising anti-inflammatory target.

## INTRODUCTION

Mastitis is one of the most common diseases in dairy farming (1). This disease not only reduces the production performance of cows but also causes significant economic losses to the breeding industry worldwide. It is generally believed that the invasion of bacteria is the main cause of mastitis, with *S. aureus* being one of the most important pathogens (2). Due to its immune evasion mechanism (3), *S. aureus* is difficult to clear once the mammary glands have been infected. Currently, in clinical practice, the treatment of *S. aureus* infection still relies mainly on antibiotics, but the widespread use of antibiotics can easily lead to problems, such as bacterial resistance and drug residues, posing a threat to food safety (4). Therefore, it is urgent to find a safe and effective treatment method.

Upregulated glycolysis is intricately associated with infection-induced inflammation, which has been observed in a variety of pathogenic infections (5–8). Hypoxic inducible factor 1α (HIF1α) plays a vital role in this metabolic shifting and regulates the host metabolism from oxidative phosphorylation to glycolysis, which is called aerobic glycolysis (9). During pathogenic infections, the HIF1α pathway is rapidly induced to meet the metabolic needs for defense. In short, inflammatory signals trigger the accumulation of HIF1α in the cytoplasm, which promotes the upregulation of glycolysis and inflammation-related genes after translocation to the nucleus (10). This process can be initiated by the activation of Toll-like receptors (TLRs) or the production of reactive oxygen species (11).

STING-mediated type I interferon signaling mainly participates in effective immune responses against viral infections (12). The role of STING during bacterial diseases is controversial, ranging from protective to detrimental effects for the host. STING is involved in diverse bacterial infections and exerts different immune responses depending on pathogens and different infectious models. Studies have demonstrated that STING plays a protective role in response to bacterial infections such as *Brucella* (13) and *Mycobacterium tuberculosis* (14). In particular, in the model of *S. aureus* cutaneous infection, activation of STING antagonized innate immunity and resulted in infection spread through decreased neutrophil recruitment and IL-1β secretion (15). However, much less is known about the biological implications of STING in host defense, specifically during mammary gland microbial infections.

Our study demonstrates that STING plays a critical role in metabolic reprogramming of mammary epithelial cells against *S. aureus* infection via mitochondrial reactive oxygen species (mROS)-HIFα signaling, which stimulates an increase in glycolysis in the host, subsequently resulting in inflammation. By inhibiting STING, it potentially reduces HIF1α-driven glycolysis by decreasing mROS production, resulting in less inflammatory damage during infection. These findings suggest a new therapeutic approach for treating *S. aureus* infections in mammary glands.

## MATERIALS AND METHODS

### Bacterial strain, cell culture, and treatment

*S. aureus* (ATCC 29213), *Streptococcus uberis* (0140J) and *Escherichia coli* (NJ-17 strain) were inoculated into tryptic soy broth, Todd-Hewitt broth, and Luria-Bertani, respectively, and incubated at 37°C in an orbital shaker to log-phase growth.

Mammary epithelial cell (MEC) line EpH4-Ev (ATCC, Manassas, VA, USA) was grown in Dulbecco’s modified Eagle’s medium (DMEM) with 10% fetal bovine serum (Gibco, USA). For intracellular *S. aureus* infection, cells were infected with *S. aureus* for 1 h, washed with PBS, and cultured with DMEM containing gentamicin (100 µg·mL^−1^) for 3 h.

### Mammary infection

The pregnant C57BL/6J wild-type (WT) and STING KO mice were infused with *S. aureus* in 50 µL (*S. aureus* grown at an OD_600_ of 0.5–0.6, about 1 × 10^9^ CFU per mL) saline into the L4 and R4 teats, and saline was given to the control group. Twenty-four hours after infection, mice were euthanized, and the mammary glands were collected aseptically and stored at −80℃ until analysis.

### RNA extraction and qPCR

Total RNA was isolated by TRIzol reagent (TaKaRa, Dalian, China) and converted into cDNA. Subsequently, qPCR assays were performed using SYBR Green (Roche, Basel, Switzerland) on an ABI Prism 7500 sequence detection system (Applied Biosystems, Waltham, MA, USA). Fold changes were calculated as threshold cycle (2^-ΔΔ*CT*^) values. The appropriate primers were used to amplify a specific fragment corresponding to gene targets as described in Table S1.

### Western blot and antibodies

Cell lysates were frozen in radioimmunoprecipitation assay buffer (Beyotime, Nantong, China) containing phenylmethylsulfonyl fluoride (Beyotime). Antibodies for Tubulin β (Bioworld), HIF1α (Cell Signaling), TMEM173/STING (Proteintech), TBK1 (Bioworld), p-TBK1 (Bioworld), IRF3 (Cell Signaling), and GM130 (Cell Signaling) were used. Signals were detected using an ECL Western blot analysis system (Tanon, Shanghai, China). Bands were quantified using ImageJ software (NIH, USA).

### Cytokine measurements

TNF-α, IL-1β, and IFN-β levels in MECs or mammary glands were quantified using enzyme-linked immunosorbent assay kits according to the manufacturer’s instructions (R&D Systems, CA, USA).

### Lactate and LDH assays

The lactate content and lactate dehydrogenase (LDH) activity were determined using commercial kits according to the manufacturer’s instructions (Solarbio, Beijing, China).

### Detection of relative enzyme activities

HK2 and PFK1 activities were measured by commercial kits (HK2 assay kit and PFK1 assay kit) according to the manufacturer’s instructions (Solarbio).

### Intracellular viable bacterial count assay

Viable bacteria were enumerated as CFU on BHI agar. CFUs were counted by the spread plate method after incubation for 12 h at 37°C. After infection with *S. aureus* at the mid-exponential phase, *S. aureus*-infected cells were washed three times with PBS containing 100 mg/mL of gentamicin, followed by gentamicin-free PBS. Equal numbers of cells were lysed with sterile triple-distilled water, and CFUs were counted by the spread plate method after incubation for 12 h at 37°C.

### Immunofluorescence staining

For the protein expression assay, cells were collected and fixed in 4% paraformaldehyde for 30 min, washed with PBS, permeabilized with 0.1% Triton-X-100 (Merck), blocked in 5% BSA (Solarbio) in PBS, and incubated with primary antibodies at 4°C overnight. Then, the cells were washed with PBS and incubated with a secondary antibody at 37°C for 1 h, followed by staining with 4′,6-diamidino-2-phenylindole (DAPI). The slides were subsequently mounted using glycerin and observed under a confocal microscope (Olympus, Tokyo, Japan).

### Detection of mROS

Intracellular mROS was stained by MitoSOX Red Mitochondrial Superoxide Indicators according to the instructions (ThermoFisher Scientific). Briefly, cells were incubated in PBS containing 5 µM MitoSOX Red Mitochondrial Superoxide Indicator for 30 min at 37°C. Emitted fluorescence (Ex/Em = 510/580) was measured. Cells were collected to be analyzed by flow cytometry (Beckman) equipped with FlowJo software.

### Statistical analyses

Statistical analyses were performed using GraphPad Prism 9 software. Sample numbers and repetitions are indicated in the figure legends. All data were analyzed using an unpaired *t* test and one-way analysis of variance (ANOVA) as indicated in the figure legends. All data are presented as means ± standard deviations (SD) or standard errors of the means (SEM). For all experiments, *P* values < 0.05 were considered significant.

## RESULTS

### *S. aureus* activates the STING signaling pathway in MECs

**Fig 1:**
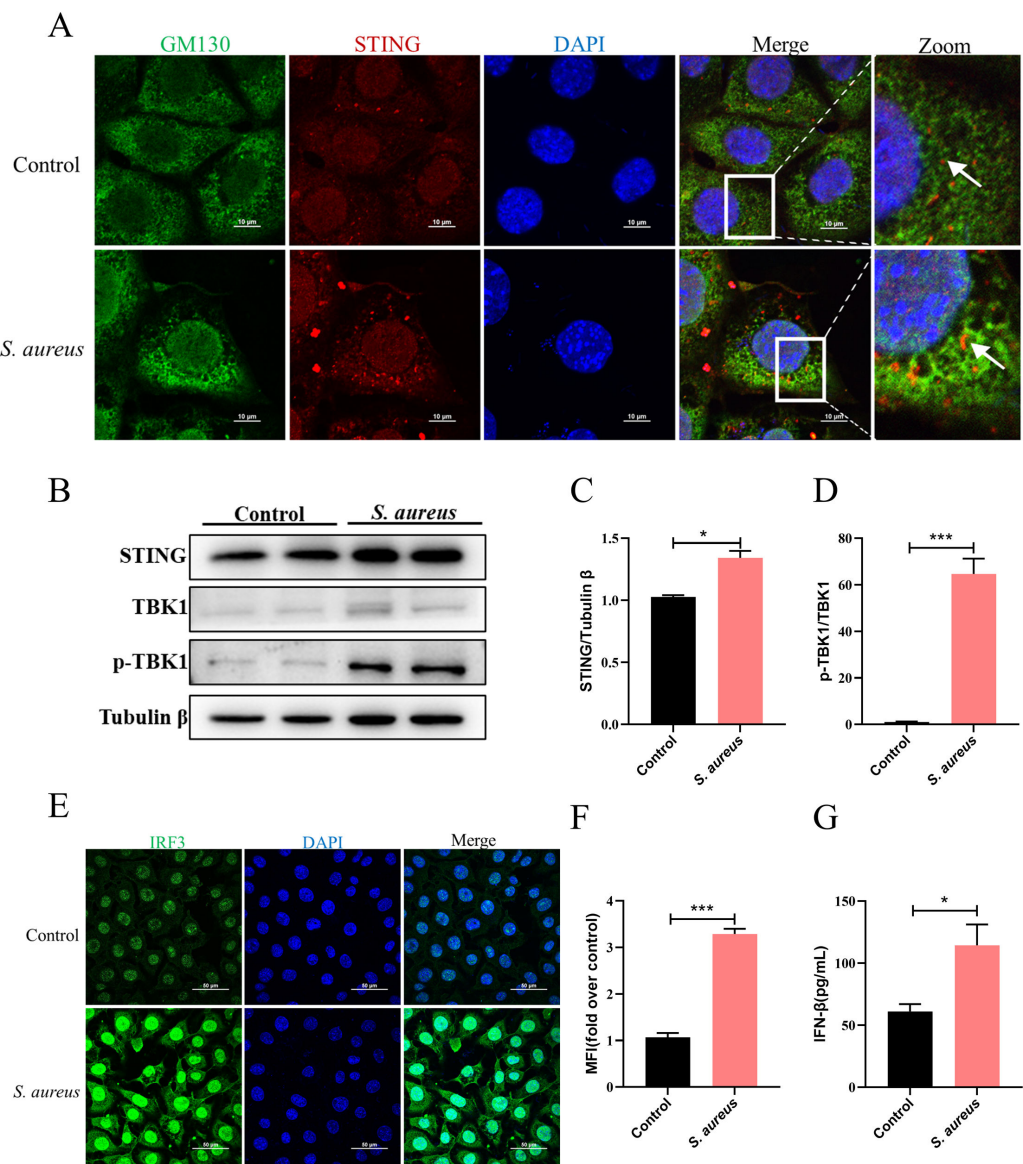
*S. aureus* activates the STING signaling pathway in MECs. (A) Confocal analysis of GM130 (green), STING (red), and DAPI (blue) in MECs. Scale bar, 10 μm. (B–D) Protein levels of STING and p-TBK1 in MECs without and with *S. aureus* infection for 4 h. (E and F) Confocal analysis of IRF3 (green) and DAPI (blue) in MECs. (G) IFN-β level in cell supernatants (*n* = 3). Data are presented as mean ± SD (*n* = 3). *P < 0.05 and ***P < 0.001, i.e., significantly different between the indicated groups by unpaired *t* test.

STING pathway in bacterial infection is complex since the protective and detrimental effects of IFN-β depend on the bacterial species and infection mode. It is unknown whether *S. aureus* activates the STING pathway in mammary epithelial cells. Confocal microscopy analysis revealed that intracellular infection of *S. aureus* increased the accumulation of STING on the Golgi apparatus (Fig. 1A). Activated STING recruited and activated TBK1, which drove IRF3 into the nucleus to induce the transcription of IFN-β. Consistently, *S. aureus* infection led to an increase in STING (Fig. 1B and C), phosphorylated TBK1 (P-TBK1) (Fig. 1D), and nuclear IRF3 expression (Fig. 1E and F). Furthermore, *S. aureus* enhanced the inflammatory response of IFN-β (Fig. 1G). Taken together, these findings suggested that *S. aureus* promoted the activation of the STING pathway in infected cells.

### STING promotes *S. aureus*-induced inflammation

**Fig 2:**
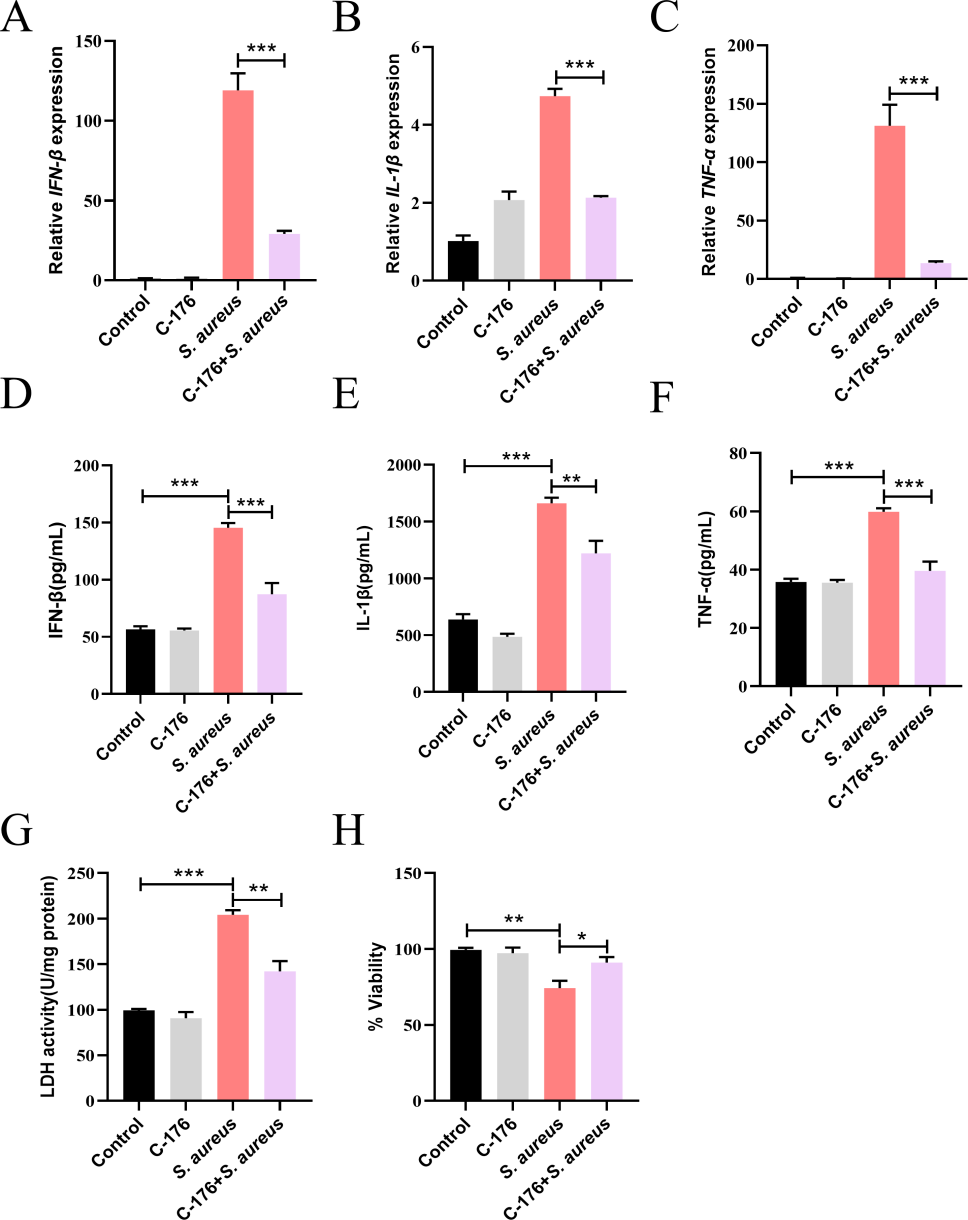
STING promotes *S. aureus*-induced inflammation. MECs were infected with *S. aureus* at an MOI of 10 and incubated at 37°C as described in Materials and Methods. (A–C) Relative expression of cytokine (IFN-β, IL-1β, and TNF-α). *n* = 6. (D–F) Cytokine (IFN-β, IL-1β, and TNF-α) levels in cell supernatants. (G) The LDH activity in cell supernatants. (H) Cell viability assessed by trypan blue exclusion. Data are presented as mean ± SEM (*n* = 3). *P < 0.05, **P < 0.01, and ***P < 0.001, i.e., significantly different between the indicated groups by one-way ANOVA.

To assess the role of STING in MECs against *S. aureus* infection, cells were pretreated with or without C-176, a STING antagonist, before *S. aureus* challenge. Compared to *S. aureus* counterparts, C-176 significantly inhibited the secretion of pro-inflammatory cytokines (IFN-β, TNF-α, and IL-1β) (Fig. 2A through F). In addition, C-176 reduced cell damage caused by *S. aureus* infection, as shown by LDH activity and cell survival assays (Fig. 2G and H). Collectively, these results suggested that STING played a pro-inflammatory role for MECs against *S. aureus* infection.

### Escalation of inflammation associated with STING relies on the mROS-HIF1α axis

**Fig 3:**
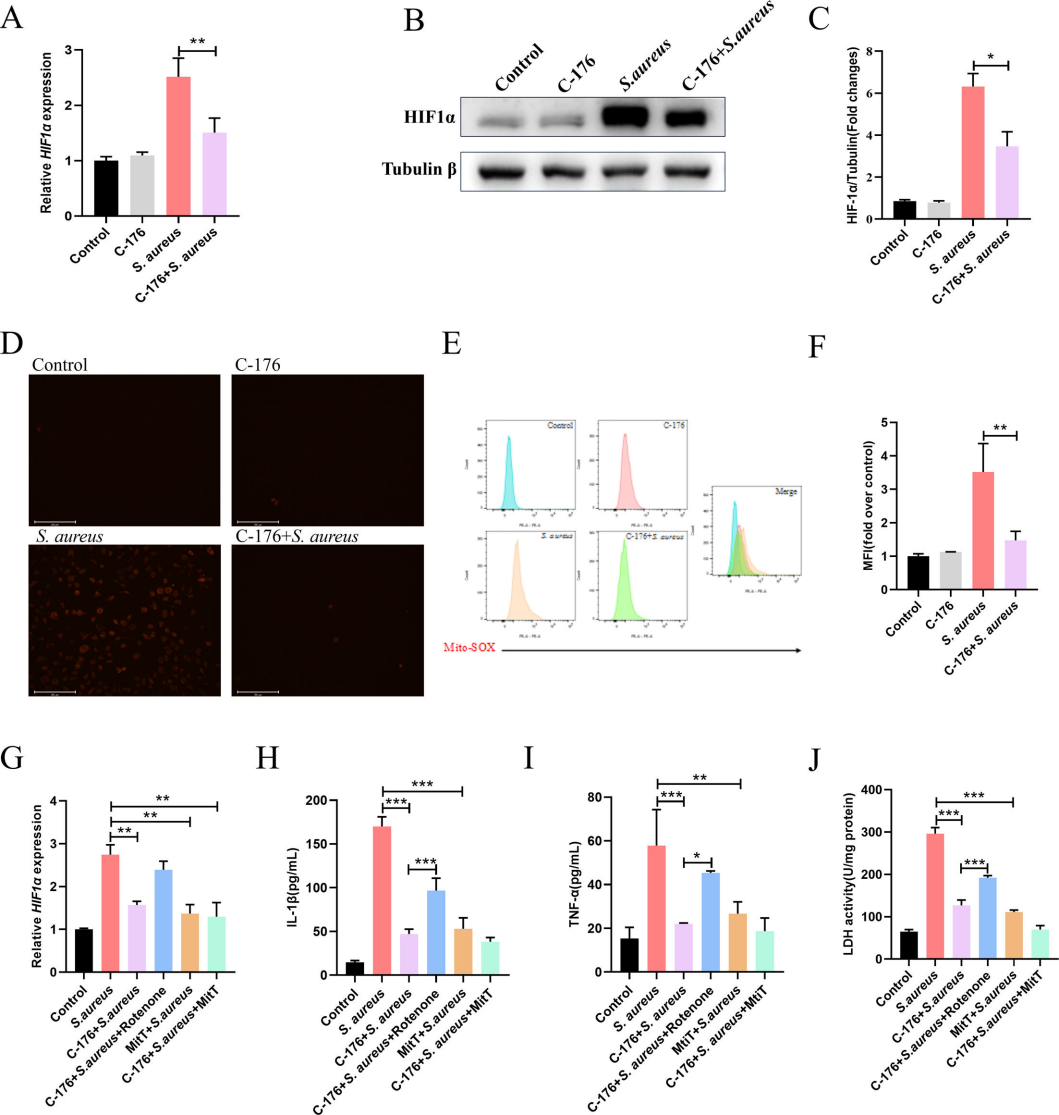
Escalation of inflammation associated with STING relies on the mROS-HIF1α axis. The cells were treated with 5 μM C-176 for 4 h, 1 μM rotenone for 1 h, and 5 mM MitT for 3 h prior to the *S. aureus* infection. (A) Relative expression of HIF1α. *n* = 6. (B and C) The protein expression levels of HIF1α. (D) Confocal analysis of mROS (red) in MECs. Scale bar, 200 μm. (E and F) Intracellular mROS content was evaluated by staining the cells (10,000 cells per sample) with Mito-SOX, followed by analysis using CellQuest Pro acquisition and FlowJo software. (G) Relative expression of HIF1α. *n* = 6. (H and I) Cytokine (IL-1β and TNF-α) levels in cell supernatants. (J) The LDH activity in cell supernatants. Data are presented as mean ± SEM (*n* = 3). *P < 0.05, **P < 0.01, and ***P < 0.001, i.e., significantly different between the indicated groups by one-way ANOVA.

Such alteration in host inflammation is regularly accompanied by metabolic changes. In that context, HIF1α arose as an important component of the cellular machinery involved in the control of cell behavior and cellular metabolism (16). Therefore, we hypothesized that HIF1α might be linked to the STING-dependent induction of the inflammatory profile. Indeed, we observed that HIF1α (Fig. 3A through C) was upregulated during *S. aureus* infection, and C-176 pretreatment reversed these changes. These findings demonstrated that STING contributed to establishing an inflammatory profile in mammary epithelial cells during *S. aureus* infection and drove a consistent expression of HIF1α.

mROS has been reported to regulate cellular functions by modulating the expression of HIF1α and STING-induced mROS production in macrophages infected with *Brucella abortus* (17). Thus, we hypothesized that STING may control the expression of HIF1α through the regulation of mROS in MECs. To test this hypothesis, we initially determined mROS formation in mammary epithelial cells following *S. aureus* infection. The results indicated that *S. aureus* infection led to an increased mROS level, and the joint treatment of C-176 significantly inhibited mROS production (Fig. 3D through F). It was reported that mROS affected HIF1α stability by inactivating PHD2 (18). Our results demonstrated that HIF1α levels were significantly increased in *S. aureus*-infected and rotenone-treated cells, compared to mock-infected cells, and this effect was counteracted by the mROS scavenger Mito-TEMPO (MitT) (Fig. 3G). Additionally, MitT reduced the expression of inflammatory cytokines IL-1β (Fig. 3H), TNF-α (Fig. 3I), and LDH activity (Fig. 3J) in supernatant, while rotenone reversed the anti-inflammatory effect of C-176. Taken together, our findings suggested that C-176 regulated HIF1α accumulation in a mROS-dependent manner.

### STING-elevated inflammation is linked with glycolysis driven by HIF1α

**Fig 4:**
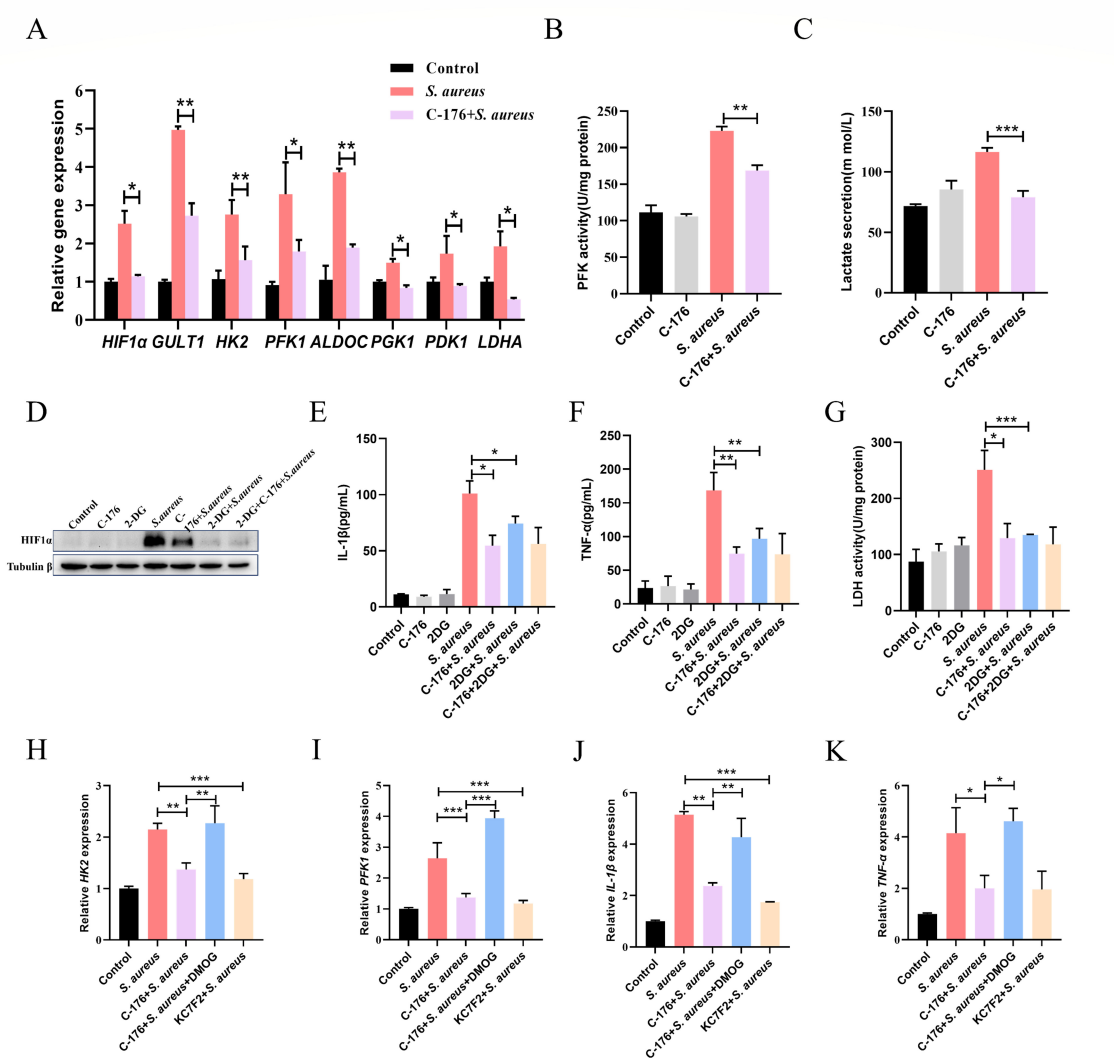
STING-elevated inflammation is linked with glycolysis driven by HIF1α. The cells were treated with 5 μM C-176 for 4 h, 5 mM 2-DG for 1 h, 50 μM DMOG or 20 μM KC7F2 for 24 h prior to the *S. aureus* infection. (A) Relative expression of the HIF1α-driven glycolysis-associated gene. *n* = 6. (B) The PFK1 activity of MECs. (C) The lactate levels in cell supernatants. (D) The protein expression levels of HIF1α. (E and F) Cytokine (IL-1β and TNF-α) levels in cell supernatants. (G) The LDH activity in cell supernatants. (H–K) Relative expression of HK2, PFK1, IL-1β, and TNF-α. *n* = 6. Data are presented as mean ± SEM (*n* = 3). *P < 0.05, **P < 0.01, and ***P < 0.001, i.e., significantly different between the indicated groups by one-way ANOVA.

C-176 pretreatment reduced HIF1α-dependent signaling of glycolysis (Fig. 4A). There was a significant difference in PFK1 (Fig. 4B) activity and secretion of lactate (Fig. 4C) after C-176 treatment in *S. aureus*-infected cells. To establish the link between HIF1α-driven glycolysis and inflammation in *S. aureus* infection, we analyzed HIF1α expression in the presence of a specific inhibitor of glycolysis (2-deoxy-D-glucose, 2-DG). As depicted, 2-DG inhibited *S. aureus*-induced HIF1α expression (Fig. 4D), IL-1β (Fig. 4E), TNF-α (Fig. 4F) levels, and LDH activity (Fig. 4G) in supernatant consistent with C-176 treatment.

To investigate the role of HIF1α in the metabolic response to *S. aureus* infection, we treated mammary epithelial cells with KC7F2, a selective inhibitor of HIF1α. Our results indicated that the expression of key glycolytic enzymes, HK2 and PFK1, was decreased in response to KC7F2 treatment (Fig. 4H and I). Moreover, inhibiting HIF1α resulted in lower levels of the inflammatory cytokines IL-1β and TNF-α (Fig. 4J and K). Conversely, treatment with DMOG, an HIF1α agonist, reversed the effects of C-176 treatment. These findings indicated that HIF1α-induced glycolysis in *S. aureus* infection promotes inflammation.

### STING promotes intracellular *S. aureus* proliferation

**Fig 5:**
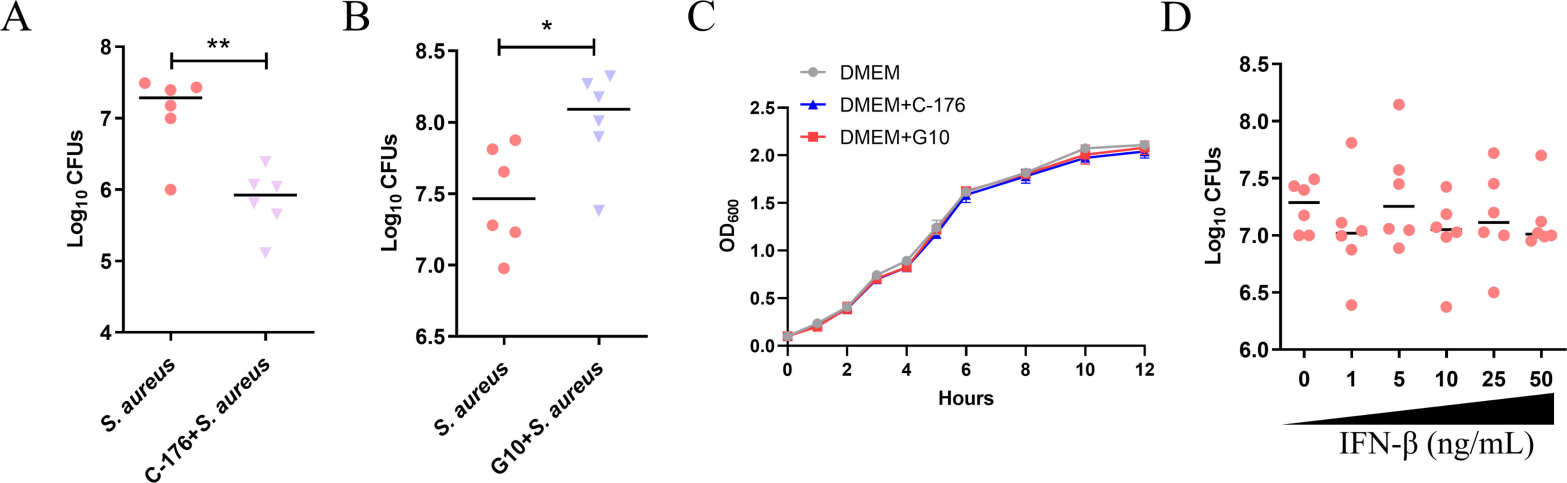
STING promotes intracellular *S. aureus* proliferation. The cells were treated with 5 μM C-176, 5 μM G10, or various concentrations of IFN-β for 4 h prior to the *S. aureus* infection. (A, B, and D) The number of *S. aureus* colonies in MECs. (C) *S. aureus* was diluted to 0.1 of OD_600_ with DMEM, cultured in a medium with C-176 or G10, and OD_600_ was determined at a specific time. Data are presented as mean ± SD (*n* = 6). *P < 0.05 and **P < 0.01, i.e., significantly different between the indicated groups by *t* test.

To explore the impact of STING on intracellular bacteria survival, MECs were treated with C-176 and G10 (an activator of STING), respectively, before *S. aureus* infection. Inhibition of STING significantly decreased bacterial accumulation within cells (Fig. 5A). On the contrary, G10 treatment promoted the proliferation of *S. aureus* in the host (Fig. 5B). Meanwhile, the direct impact of C-176 and G10 on the proliferation of *S. aureus* was negligible (Fig. 5C). What is more, exogenous addition of IFN-β made no difference for intracellular proliferation, although we increased the concentration of IFN-β (Fig. 5D).

### STING exacerbates mastitis via the HIF1α-glycolysis axis

**Fig 6:**
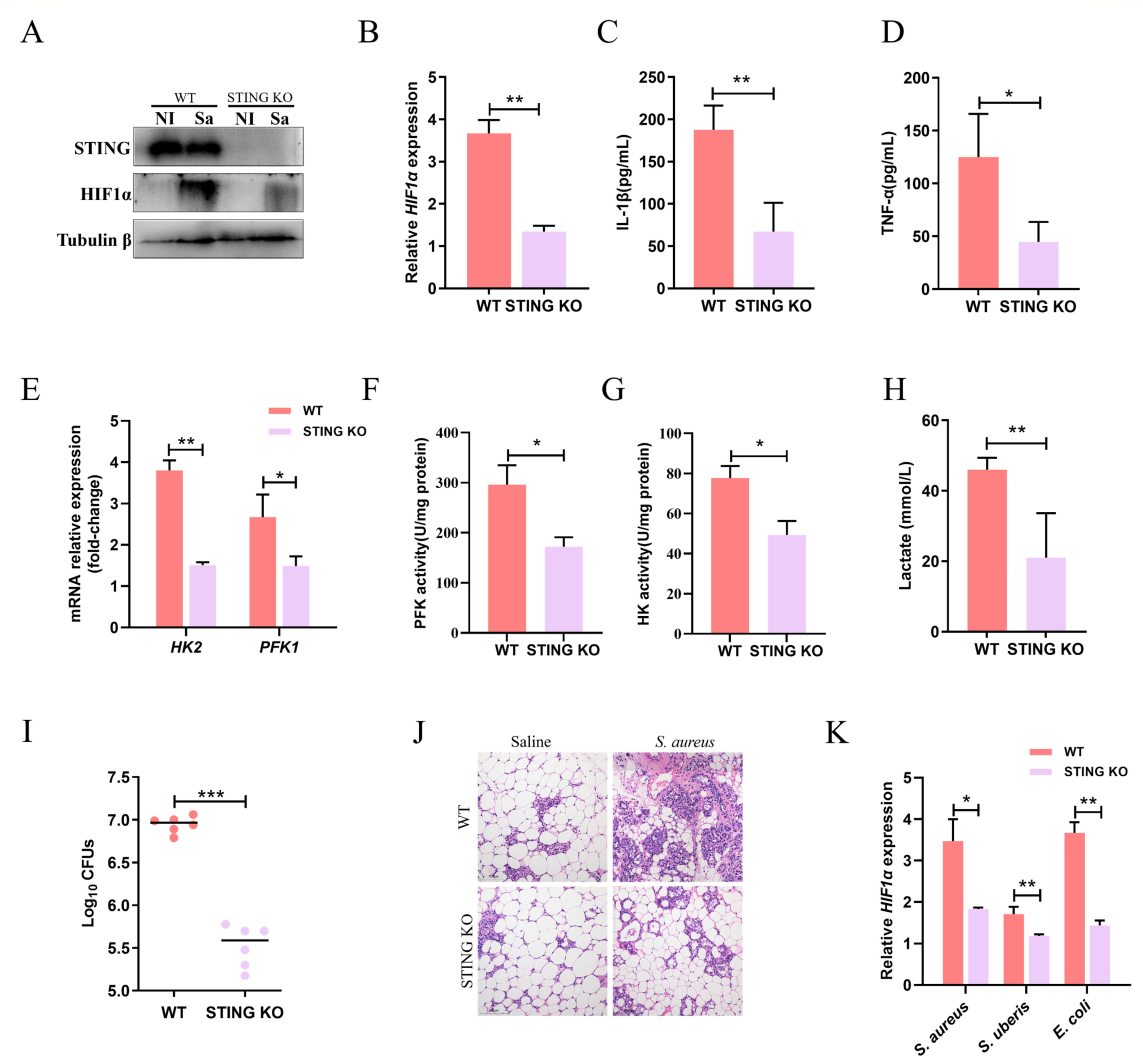
STING exacerbates mastitis via the HIF1α-glycolysis axis. At 72 h post-parturition, the C57BL/6 mice and STING KO mice were infused with *S. aureus* in 50 μL of sterile saline into the left fourth (L4) and right fourth (R4) teats. At 24 h post-*S. aureus* infusion, mammary glands were collected for analysis. (A and B) Western blot detection and quantitative analysis of HIF1α. (C and D) Cytokine (IL-1β and TNF-α) levels in mammary gland supernatants. *n* = 6. (E) Relative expression of *HK2* and *PFK1*. *n* = 6. (F and G) The PFK1 and HK activity of mammary gland supernatants. *n* = 6. (H) The lactate levels in mammary gland supernatants. (I) The number of *S. aureus* colonies in mammary glands. *n* = 6. (J) H&E staining of sections from infected mammary glands. (K) Relative expression of HIF1α. *n* = 6. Data are presented as mean ± SD (*n* = 3, unless otherwise indicated). *P < 0.05, **P < 0.01, and ***P < 0.001, i.e., significantly different between the indicated groups by unpaired *t* test.

To investigate the *in vivo* significance of STING in *S. aureus* infection, WT and STING KO mice were challenged with *S. aureus* for 24 h. Compared to WT mammary glands, STING KO mice showed decreased expression of HIF1α (Fig. 6A and B) and significantly less production of IL-1β (Fig. 6C) and TNF-α (Fig. 6D). Consistent with the *in vitro* results, infected mammary glands of STING KO mice exhibited reduced mRNA levels of HIF1α-target glycolytic genes (*HK2* and *PFK1*) (Fig. 6E), decreased activities of key glycolytic enzymes (HK2 and PFK1) (Fig. 6F and G), and diminished lactate secretion (Fig. 6H). At the same time, the deficiency of STING restricted the proliferation of *S. aureus* in the mammary glands of infected mice (Fig. 6I). Although the number of immune cells in mammary gland alveoli increased with *S. aureus* infection, STING KO mice reversed this pathological change (Fig. 6J).

To further assess the potential of STING as a therapeutic target for mastitis, we tested its efficacy against two other pathogens known to cause mastitis, *S. uberis* and *E. coli*, in addition to *S. aureus*. At the transcriptional level, STING KO mice significantly reduced the expression of HIF1α genes (Fig. 6K). Taken together, our findings suggested that STING could serve as a potential target for the treatment of mastitis.

## DISCUSSION

*S. aureus* is a common pathogen in humans and livestock associated with persistent infections. Although antibiotics restrict bacterial proliferation, severe drug resistance remains a serious problem facing the dairy industry. Therefore, the need for new therapeutic strategies has become more urgent.

Beyond sensing viruses, the role of STING during bacterial infection is elusive, ranging from protective to detrimental effects (19). Furthermore, the biological implications of STING in *S. aureus* mammary gland infection have not been explored. We proved that *S. aureus* infection activated the STING-TBK1-IRF3 pathway and induced the expression of IFN-β. Inhibition of STING limits the inflammatory responses and damage in MECS caused by *S. aureus* infection, which has sparked our interest in investigating whether STING could be a potential target for the treatment of bacterial infections.

Host cells reprogram their metabolic pathways against pathogenic infection, and these changes are directly linked to pathogen growth or restriction (20). It was previously demonstrated that *S. uberis* infection caused metabolic and inflammatory transition in MECs and mammary glands. Inflammatory responses could be restrained by regulating this metabolic reprogramming through taurine (21, 22), 2-DG, and PFK15 (23). Based on these, we assume that STING regulates host metabolic reprogramming through HIF1α, a major transcription factor activated in response to infection, enabling the cells to meet the increased metabolic demands (24). Mechanistically, HIF1α stability is controlled by the hydroxylation of proline residues promoted by prolyl hydroxylase (PHD) (25). Succinate excess impairs PHD activity, leading to HIF1α stabilization and activation, which facilitates the metabolic shift from OXPHOS to glycolysis (26). In addition, accumulated succinate can be oxidized to fumarate by succinate dehydrogenase, driving mitochondrial ROS production through reverse electron transport from complex II to complex I, leading to mROS-dependent HIF1α stabilization (27).

In this study, we demonstrate that HIF1α expression is dependent on mROS. Inhibition of STING by C-176 reduces mROS production and consequently, HIF1α expression. During *S. aureus* infection, MECs upregulate ROS to fight the invading pathogen. However, excessive ROS can activate HIF1α, resulting in severe inflammation. This ROS-HIF1α loop is observed in other bacterial infections such as *Helicobacter pylori* and has been linked to cancer pathogenesis (28). Treatment with MitT, which disrupts the mROS-HIF1α loop, reduces inflammation and damage caused by *S. aureus*. Our results suggest that reducing intracellular mROS levels inhibits HIF1α-induced inflammatory responses in *S. aureus*-infected MECs.

Under certain inflammatory conditions, eukaryotic cells can shift their main metabolic pathway from mitochondrial respiration to glycolysis to maintain ATP levels (29). According to reports, the level of glycolysis is closely related to the development of renal fibrosis and may be crucial for the progression of chronic kidney disease throughout the entire course of the disease (30). As is well known, hypoxia-induced glycolysis is regulated by multiple pathways (31–33), and exploring whether the STING pathway is involved will be an interesting matter. In a study of non-small cell lung cancer, glycolysis enhances the anti-tumor immune response mediated by dendritic cells through STING phosphorylation activation. Furthermore, STING agonist 2, 3-cGAMP promotes glycolysis to regulate intestinal homeostasis in the host (34). It follows that the activation of STING is associated with glycolysis, which is consistent with what we have observed: C-176 inhibits host-enhanced glycolysis caused by *S. aureus* infection.

It is currently unclear what causes *S. aureus* to activate the STING pathway in MECs. The c-di-AMP released from *S. aureus* biofilms activates STING to upregulate the expression of IFN-I in macrophages (35). The STING-dependent IFN-I response promotes macrophage polarization to an anti-inflammatory phenotype, resulting in impaired *S. aureus* clearance and exacerbated infectious outcomes (35). However, we found that exogenous IFN-β had no effect on the proliferation of *S. aureus* in MECs, which suggests that the effect of STING on intracellular proliferation of *S. aureus* is related to its regulation of host metabolism. Our previous research found that the proliferation of *S. aureus* in MECs was related to glucose concentration (23). In addition, the STING pathway can be activated by sensing *S. aureus* DNA in response to live *S. aureus*. In the early stage of *S. aureus* infection, both TLR and STING pathway are activated by *S. aureus* but play the opposite role in the host immune response (15). TLR signal transduction restricts infection, while STING enhances bacterial growth. Previous studies have reported that the STING pathway is involved in macrophage necrosis in an IFN-I-dependent manner (36, 37). However, in the context of *S. aureus* pneumonia, STING promotes the restriction of *S. aureus* infection and protects the structure and function of the lungs by inhibiting macrophage necrosis (38). According to these studies, IFN-I induced by the STING pathway seems to be related to the immune escape of *S. aureus*, and the activation of STING is conducive to controlling *S. aureus* infection in the lungs. Briefly, the role of STING in bacterial infections is intricate, and further research is needed on its role in mouse mammary gland infections.

Although the inhibition of STING has an anti-inflammatory effect during *S. aureus* infection, there are still some limitations in this study. Our results were simply validated in bovine epithelial cells (data not shown) but not in dairy cows. At the same time, the effect of inhibiting STING or glycolysis on mammary gland inflammation against persistent and recurrent *S. aureus* infection is also unknown. We will explore this in detail in future work.

**Fig 7:**
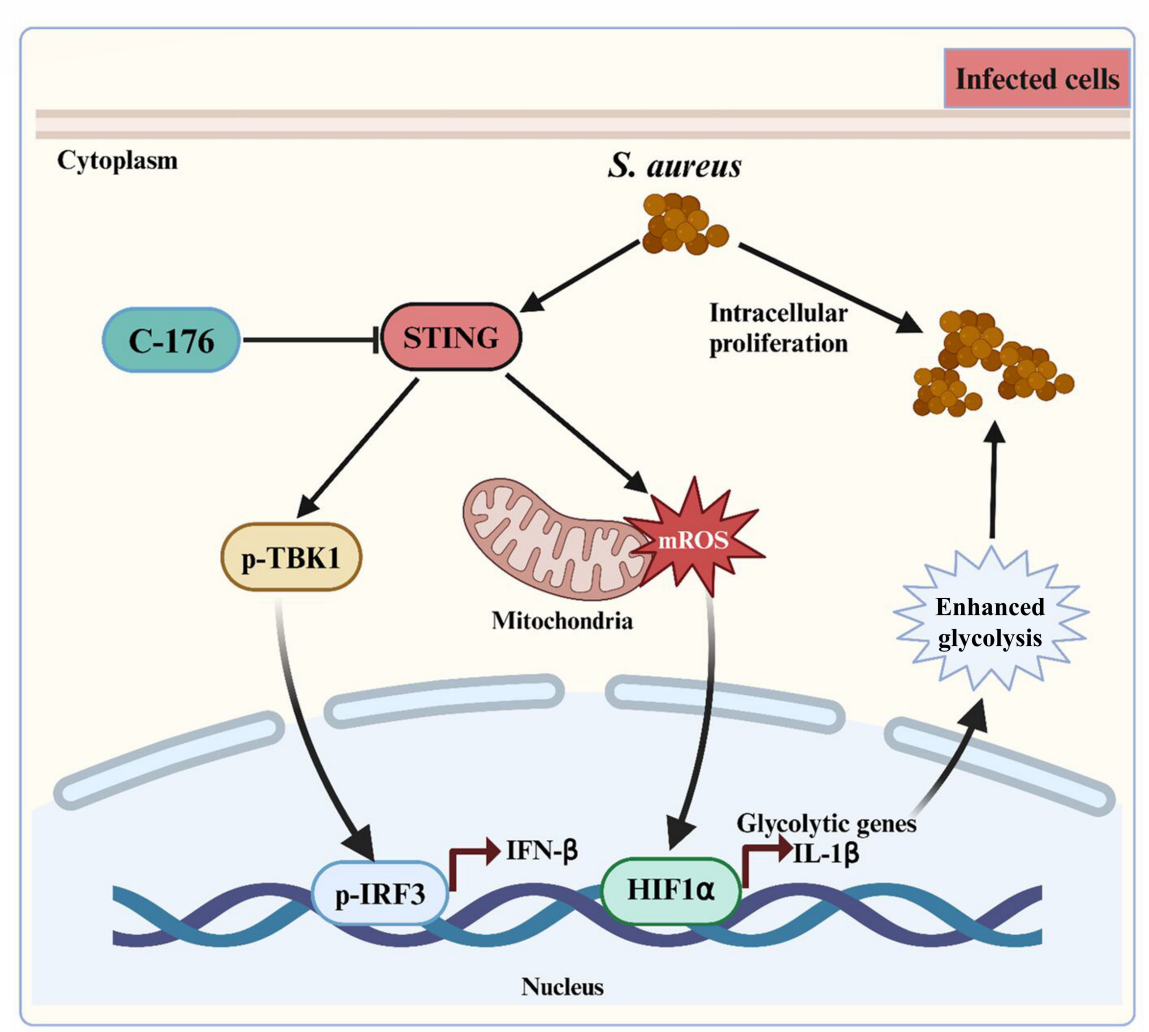
STING facilitates *S. aureus*-induced mastitis via the mROS-HIF1α-glycolysis axis.

In summary, our research indicates that inhibiting STING can play a significant role in reducing mROS-HIF1α-driven glycolysis, which can, in turn, decrease bacterial load and inflammation associated with intracellular *S. aureus* infection (Fig. 7). Inhibiting STING could be a safe and effective strategy for developing new anti-*S*. *aureus* therapies.

## SUPPLEMENTAL MATERIAL

Table S1 iai.00138-25-s0001.docx The target genes and primers for qPCR.

ASM does not own the copyrights to Supplemental Material that may be linked to, or accessed through, an article. The authors have granted ASM a non-exclusive, world-wide license to publish the Supplemental Material files. Please contact the corresponding author directly for reuse.
