# Supplementary material for: STING contributes to the inflammation and proliferation of Staphylococcus aureus via mitochondrial reactive oxygen species–hypoxic inducible factor 1α axis in epithelial cells
Source: Infect Immun. 2025 May 19;93(6):e00138-25. doi: 10.1128/iai.00138-25 (PMC12150761; doi:10.1128/iai.00138-25)
Supplement: Table S1 — The target genes and primers for qPCR. [file iai.00138-25-s0001.docx]

| Gene | Forward | Reverse |
| --- | --- | --- |
| *Hif1α* | ACCTTCATCGGAAACTCCAAA | CTGTTAGGCTGGGAAAAGTTA |
| *Glut1* | CAGTTCGGCTATAACACTGGT | GCCCCCGACAGAGAAGATG |
| *HK2* | CTAAGGGGTTCAAGTCCAGTG | AGACCAATCTCGCAGTTCTGA |
| *PFK1* | TGTGGTCCGAGTTGGTATCTT | GCACTTCCAATCACTGTGCC |
| *LDHA* | TGTCTCCAGCAAAGACTACTG | GACTGTACTTGACAATGTTGG |
| *ALDOC* | AGAAGGAGTTGTCGGATATTG | TTCTCCACCCCAATTTGGCTC |
| *PGK1* | ATGTCGCTTTCCAACAAGCTG | GCTCCATTGTCCAAGCAGAAT |
| *PDK1* | GGACTTCGGGTCAGTGAATGC | TCCTGAGAAGATTGTCGGGGA |
| *IL-1β* | GCAACTGTTCCTGAACTCAAC | ATCTTTTGGGGTCCGTCAACT |
| *TNFα* | GACGTGGAACTGGCAGAAGA | TTGGTGGTTTGTGAGTGTGAG |
| *IFN-β* | CAGCTCCAAGAAAGGACGAA | GGCAGTGTAACTCTTCTGCAT |
| *β-actin* | GGCTGTATTCCCCTCCATCG | CCAGTTGGTAACAATGCCATG |

TABLE S1 The target genes and primers for qPCR.
